# Application of the Rubber Dam

**Published:** 1892-12

**Authors:** Theo F. Chupein

**Affiliations:** Philadelphia.


ARTICLE III.

### APPLICATION OF THE RUBBER DAM.

BY THEO F. CHUPEIN, PHILADELPHIA.

Read before the Southern Dental Association.

I do not know that the few scattering items which I offer can be classed under the appellation of a paper, but as your chairman requested the bringing out of anything new, I present to your consideration such items as have been suggested during the year.

Cases occur in practice almost daily where this invaluable adjunct of dentistry becomes useless, because it cannot be properly applied. As a typical case, let us suppose an upper molar to be extensively decayed on its mesio-masticating aspect, while the tooth is intact on its distal surface. The decay has progressed so far above the gum where the tooth is decayed, that in order to prepare the cuncal margin the absorption of the gum is necessary. It is generally known that this may be effectually done by packing gutta percha between the tooth where we wish to effect such absorption. But though we may effectually produce this absorption at the point where the tooth is decayed, the gum adheres to the neck of the tooth in its normal position at all other points, and if we wish to apply the dam in order to aid us in filling such a cavity, we are thwarted on this account. To overcome this difficulty, we proceeded as follows :

Pack gutta percha at the distal as well as at the mesial surface of the tooth; when the gum is thus forced away from these surfaces, remove the gutta percha and mop gilling thread or ligature silk two or three times around the tooth, pushing this up well on the neck of the tooth, then tie and replace the gutta-percha at both proximate surfaces. Dismiss the case for a day or two, when the dam may be applied without the least difficulty, the gums having been forced well away by the gutta percha at its proximate surfaces, and by the ligature at other surfaces. Try it, and rejoice.

THE OIL OF CAJEPUT AND GUTTA PERCHA.

It frequently happens that there are cavities where the application of the rubber dam is inadmissable, but such cavities cannot be kept dry. Dr. W. H. Roop contends that by means of a very light film of the oil of cajeput, gutta percha may be packed into a wet cavity and made to adhere, either for a temporary purpose previous to filling with gold, or for a permanent filling where the material is not subject to the attendance of mastication.

The cavity is prepared under such circumstances without wounding the gum, the cavity made as dry as it may be with any absorbent that is used, and a piece of gutta percha just sufficient to fill the cavity is softened, and before it is introduced it is moistened with a film of the oil of cajeput, using no more on the pellet than what may be transferred to it from what adheres to the cork of the vial. To substantiate this Dr. Roop, at one of the meetings of the Pennsylvania Association of Dental Surgeons, procured a tumbler of water; he then softened a small pellet of gutta percha, coated this slightly with a film of the oil of cajeput, and with an ordinary ball burnisher pressed this against the inside of the tumbler beneath the water. The gutta percha adhered to the glass with sufficient force to not only hold its place but to require some force to dislodge it.

Dr. Roop also presented samples of gutta percha which softened readily by the hot water bath, yet attained a sufficient hardness to enable it to be used on the masticating surfaces of the teeth.

### ANNEALING GOLD FILLINGS.

Large contour gold fillings frequently get their corners chipped or broken off by accident or by too severe use, yet sufficient remaining in the tooth as to only require an addition made to the remainder to restore it to its former condition. This was formerly done by drilling pits into the filling and starting the new gold from these pits. Several devices within the past year have been suggested to so anneal the gold in the tooth so as to drive off all the moisture they may contain, when by simply roughening the surface of the filling, fresh gold can be readily started on the defaced plug.

One of the least expensive, readily made and most effective of these appliances has been suggested by Dr. D. V. Beacock, of Brockville, Canada. It consists merely of a small glass pipet. This is filled with cotton lampwick, with the merest end of the wick protruding from the glass pipet; by placing this in a vessel of alcohol and pressing the rubber bulb, and then removing the pressure, a sufficient quantity of the alcohol is drawn into the pipet to moisten the wick. The end may now be ignited, when it burns with a minute, almost imperceptible blaze. By using this carefully next the filling by degrees, the patient is not subjected to much discomfort, and the filling made so dry that fresh gold readily adheres to the old filling by simply roughening the surface.

The device was illustrated in the May (1892) number of the "Dental Office and Laboratory."

### NEUTRALIZING DECAYED DENTINE AND SENSITIVE DENTINE.

We have been very successful in overcoming sensitive dentine in cases of exalted sensibility, by the use of a pencil of nitrate of silver applied to the decay. This agent blackens the surface to which it is applied; but when the cavity is prepared and shaped for filling, the discoloration does not seem to permeate to any considerable depth, so that it is entirely removed by the excavating or cutting instruments, and the preparation of the cavity is almost painless.

We have also tried it to neutralize decay in large cavities in the sixth year molars of children's teeth, where these teeth have had such large cavities that if all the decayed dentine were removed, an exposure of the pulp would be the consequence.

We applied the nitrate of silver in such places in powder, rubbing it into the disorganized tissue and filling over this with gutta percha. As far as we know to the contrary, the operations have been successful.—*Southern Dental Journal.*

